# Single-Solvent Fractionation and Electro-Spinning Neat Softwood Kraft Lignin

**DOI:** 10.1021/acsabm.3c00278

**Published:** 2023-07-31

**Authors:** Bongkot Hararak, Inam Khan, Gerard F. Fernando

**Affiliations:** Sensors and Composites Group, School of Metallurgy and Materials, University of Birmingham, Edgbaston, Birmingham B15 2TT, United Kingdom

**Keywords:** softwood Kraft lignin, electro-spinning, acetone-soluble lignin, fractionation, oxidation, carbonization, characterization, carbon fibers

## Abstract

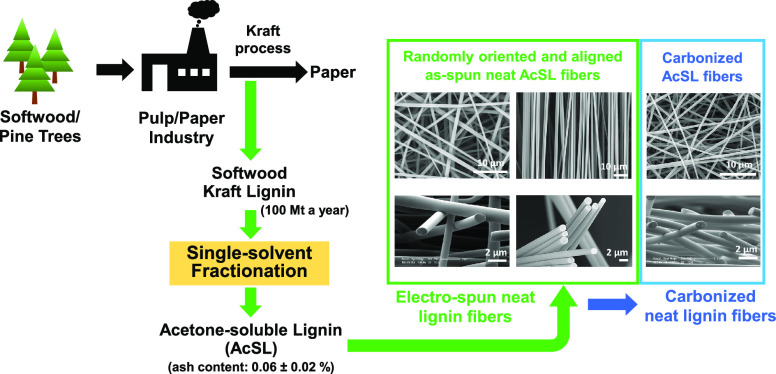

This paper reports on the production of electro-spun nanofibers from softwood Kraft lignin without the need for polymer blending and/or chemical modification. Commercially available softwood Kraft lignin was fractionated using acetone. The acetone-soluble lignin (AcSL) had an ash content of 0.06 wt %, a weight average molecular weight of 4250 g·mol^–1^ along with the polydispersity index of 1.73. The corresponding values for as-received lignin (ARL) were 1.20 wt %, 6000 g·mol^–1^, and 2.22, respectively. The AcS was dissolved in a binary solvent consisting of acetone, and dimethyl sulfoxide (2:1, v/v) was selected for dissolving the AcSL. Conventional and custom-designed grounded electrode configurations were used to produce electro-spun neat lignin fibers that were randomly oriented or highly aligned, respectively. The diameter of the electro-spun fibers ranged from 1.12 to 1.46 μm. After vacuum drying at 140 °C for 6 h to remove the solvents and oxidation at 250 °C, the fibers were carbonized at 1000, 1200, and 1500 °C for 1 h. The carbonized fibers were unfused and void-free with an average diameter of 500 nm. Raman spectroscopy, scanning electron microscopy, and image analysis were used to characterize the carbonized fibers.

## Introduction

Lignin is a biopolymer that is present in terrestrial plants, and it is the second most abundant renewable polymer on Earth, after cellulose.^1,2^ It has the highest aromatic content of all naturally occurring polymers.^3,4^ Lignin is amorphous, and it is made up of three primary aromatic monomeric units, namely, *p*-coumaryl, coniferyl, and sinapyl mono-lignols. The proportion of each monomeric unit in lignin is said to be influenced by its biomass species such as softwood (gymnosperms), hardwood (angiosperms), or herbaceous plants (graminoid).^5,6^ The *p*-coumaryl, coniferyl, and sinapyl mono-lignols polymerize to form lignin units that are referred to as *p*-hydroxyphenyl (H), guaiacyl (G), and syringyl (S).^5,6^ Softwood is reported to be composed of primarily guaiacyl units, while hardwood is made up of primarily syringyl and guaiacyl units.^5,7^ Although consensus has not been reached, recent studies have provided evidence to show that the structure of softwood is more linear^8,9^ than those proposed earlier.^10,11^

The primary commercial source of lignin is from the paper and pulping industries that generate approximately 100 million tons annually as a waste by-product.^12,13^ Lignin is generally classified according to its method of extraction, and these include lignosulphonates, Kraft, and organosolv lignins. The Kraft process is the dominant method that is used currently in the pulp and paper industry. Selected properties of commercially available lignin are presented in Table 1 including details of the origin of the plant, extraction process, weight average molecular weight (*M*_w_), polydispersity index (PDI), ash content, acid-soluble and acid-insoluble lignins, glass transition temperature (*T*_g_), and degradation temperature (*T*_DTG_). The molecular weight of lignin is in the range 1000–10,000 g·mol^–1^ with a polydispersity index (PDI) range between 2 and 9; this is dependent on the extraction procedures deployed along with the severity of the chemical used.

**Table 1:** Selected Properties of Selected Commercially Available Lignins

sample type	origin	extraction process	*M*_w_ (g·mol^–1^)	PDI	ash content (%)	acid-soluble lignin (%)	acid-insoluble lignin (%)	*T*_g_ (°C)	*T*_DTG_ (°C)	reference
lignosulfonate (water-soluble lignin)	softwood, hardwood	Sulfite	15,000–50,000	6–8	4–8	NA	NA	130	300	(6,14−16)
Indulin AT	softwood	Kraft	6000–8000	4–9	2–4	4.1	88.8	132	378	(17,18)
BioChoice lignin	softwood	Kraft and LignoBoost^a^	5200–6700	3–7	1.36	5.4	91.1	147	390	(17,19)
InnoForce lignin	softwood	Kraft and LignoForce^b^	6000	3–4	0.1–1.5	N/A	N/A	160	350	(20−22)
Alcell lignin	hardwood	organosolv	1300–3900	2	0.1	0.4	96.1	70–108	350	(23−28)
Protobind 2400	wheat straw	alkaline	2000–5000	3–4	1–1.6	9	79	59	372	(18)

a LignoBoost: In this process, the black liquor is acidified by carbon dioxide between 60 and 80 °C and a pH of 9.5. The filtrated lignin is redispersed in water and reprecipitated with H_2_SO_4_.

b LignoForce: Here, the oxidation of black liquor is carried out using oxygen at 75–80 °C until the sulfide content is reduced, prior to precipitation by carbon dioxide, and until the pH drops to 9.5. Then, the slurry containing the precipitated lignin is coagulated by mixing at the lower temperature between 60 and 65 °C.

In the context of manufacturing synthetic fibers, the molecular weight range stated in Table 1 for lignin is relatively low in comparison. Hence, in production processes such as melt-spinning,^29−32^ dry-spinning,^33−36^ wet-spinning,^37−41^ and melt-blowing^42^ of lignin, blending it with other polymers or chemical modification is required prior to the production of fibers. This is also true for electro-spinning lignin.^43−49^ The volume-loading of the polymer blend, its volatility, chemical compatibility with lignin, and volatilization during heat treatment will influence the desired mechanical properties.^31,32^ The PDI of lignin (2–9) is also relatively broad when compared to synthetic polymers (1.5–2) that are used for manufacturing fibers. In general, a lower PDI is favored with regard to processability and the production of fibers.^50^ Pretreatment strategies to reduce the heterogeneity in lignin include fractionation,^45,46,51,52^ membrane separation,^53,54^ segmented continuous flow,^55^ combinations involving organic solvents,^56−58^ and sequential acid fractionation.^59,60^ The ash content seen in Table 1 for commercially available lignin is in the range of 0.1–4%. It is known that impurities of this nature have a negative influence on the production fibers and they are also known to influence the pyrolysis kinetics.^67−69^ It is a common laboratory practice to reduce the carbohydrate^61^ and ash contents^17^ in lignin by washing it with a dilute acid.^62−66^

Although the feasibility of melt-spinning lignin has been demonstrated,^57,63,70^ the majority of the reports on spinning lignin are based on processing lignin solution including, wet-spinning,^37,71^ dry-spinning,^33−35^ and electro-spinning.^45,46,72−74^ Electro-spinning of lignin fibers without blending has been reported for hardwood organosolv lignin.^25,75^ However, this is not the case for softwood lignin where the pretreatments mentioned previously such as chemical modification^45,46,72−74,76^ and blending^43,45−49,64,77−79^ are carried out to improve its viscoelasticity properties and processability. Polymers that have been used previously for blending lignin include poly(ethylene oxide),^43,45,46,64^ polyacrylonitrile,^77,78^ and polyvinyl alcohol.^47−49,79^ A variety of solvents can be used to dissolve lignin including, *N*-dimethylformamide (DMF), dimethylacetamide (DMAc), tetrahydrofuran (THF), dimethyl sulfoxide (DMSO), and ionic liquids. DMF is used commonly to prepare lignin solutions^43,45,46,64,72,79^ due to its solubility and electrical conductivity.^43,46,72^

At the time of writing, the authors were not aware of any previous publications where softwood Kraft lignin was electro-spun without blending or chemical modification. In the current work, a procedure for electro-spinning softwood Kraft lignin without blending with polymers or other types of lignin was demonstrated for the first time. The as-received softwood Kraft lignin (BioChoice lignin) was characterized using conventional analytical techniques. The predried lignin was fractionated using acetone. The ash content in the acetone-soluble and acetone-insoluble fractions were determined and compared with acid-washed lignin. The properties mentioned in Table 1 were determined. Acetone-soluble lignin (AcSL) was dissolved in a 2:1 volume ratio of acetone:DMSO. Randomly oriented electro-spun fibers of AcSL were produced in addition to highly aligned electro-spun fibers using a modified pair of parallel grounded electrodes made from graphite. The graphite rig with the electro-spun fibers enabled heat treatment of the assembly without dislodging the fibers. A heat treatment regime was developed to evaporate the solvent from the electro-spun lignin fibers prior to thermo-stabilization at 250 °C in air. This was followed by carbonization in nitrogen gas at 1000, 1200, and 1500 °C for 1 h. The surface and cross-sectional morphologies of electro-spun and carbonized fibers were determined using a scanning electron microscope (SEM). Raman spectroscopy was used to characterize the carbonized fibers.

## Experimental Section

### Materials

Softwood Kraft lignin (Domtar’s BioChoice lignin) used in this study was purchased from distributor, UMP, Finland. AR-certified acetone was purchased from Fisher Scientific, UK. AR-certified hydrochloric acid (HCl), dimethyl sulfoxide (DMSO), and Potassium bromide (KBr) were obtained from Sigma-Aldrich, UK.

### Fractionation of As-Received Lignin and Treatment with Acidified Water

Prior to fractionation, the as-received BioChoice lignin (ARL) was dried in a vacuum oven (Model OVA031, Fistreem Vacuum Oven, UK) at 80 °C for 5 h. The predried lignin sample was fractionated using acetone where the mass-to-volume ratio (w/v) of lignin and acetone was 1:15, respectively. The fractionation was performed at 55 °C for 5 h under an argon atmosphere where the flow rate was set at 50 mL·min^–1^. The solution was filtered using a Buchner funnel and glass fiber filter with a 1 μm pore diameter (Whatman glass microfiber, Grade GF/B, Sigma-Aldrich, UK). Acetone in the solute was removed using a rotary evaporator (Buchi Rotavapor-R, Brinkman, Switzerland) equipped with a vacuum pump (Vacuubrand MD 1C VARIO with CVC3000 vacuum controller, Germany) under a reduced pressure of 300 mbar. The water bath was maintained at 53 °C ± 2 °C. After this period, the soluble and insoluble fractions were dried in a vacuum oven at 80 °C for 6 h. The soluble and insoluble fractions were coded as acetone-soluble lignin (AcSL) and acetone-insoluble lignin (AcIL), respectively.

Solvent fractionation and acid-washing methods were employed to compare the effectiveness of each method in removing the inorganic content from ARL. Hydrochloric acid was used for acid-washing the ARL. The ARL was predried in a vacuum oven (Model OVA031, Fistreem Vacuum Oven, UK) at 80 °C for 6 h prior to treating it with acidified water. A set of Taguchi-based experiments were carried out to determine the optimum treatment condition to minimize the inorganic content. The optimum condition for reducing the inorganic content in the lignin was found to be: (i) treating it with acidified-water at pH 2, (ii) treatment time for 15 min, and (iii) three sequential acid-washings. The acidified water and lignin were stirred using an overhead stirrer (Pro40 Digital Overhead Stirrer, SciQuip, UK). The overhead stirrer consisting of a variable-speed motor, which was attached to a polytetrafluoroethylene (PTFE) shaft with a PTFE-coated paddle, and it was operated at 200 rpm. After treatment with the acidified water, the lignin suspension was filtrated using 1 μm glass filter (Whatman glass microfibre, Grade GF/B, Sigma-Aldrich, UK) and washed several times with deionized water until the lignin suspension reached pH 7. The lignin was dried in a vacuum oven at 80 °C for 6 h and stored in an airtight container until required.

### Characterizations of ARL, AcSL, and AcIL

#### Ash Content

The ash content was determined in accordance with TAPPI T211 om-02 standard where the lignin was combusted at 525 °C ± 25 °C^80^ in a muffle furnace (Carbolite RHF16, UK). The ash content was determined using eq 1:
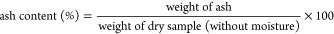
1

#### Gel Permeation Chromatography

The molecular weight and polydispersity index of the AcSL and AcIL samples were determined by using gel permeation chromatography (Agilent 1260 Infinity II Multi-Detector). This system was equipped with PLgel 5 μm mixed D columns (300 × 7.5 mm) and a PLgel 5 μm guard column. The lignins were dissolved in DMF at a concentration of 0.1 mg·ml^–1^ and filtered through 0.22 μm nylon filter. The injected volume of the lignin solution was 80 μL.

#### Quantitative Carbon Nuclear Magnetic Resonance (13C NMR) Spectroscopy

The acetylation of lignin was undertaken prior to conducting carbon nuclear magnetic resonance (^13^C NMR) analyses. The lignin was acetylated using the procedure described in the literature.^81^ The acetylated lignin was dried in a vacuum oven at 80 °C for 6 h. The quantitative ^13^C NMR spectra of lignin were obtained using predried lignin, with and without acetylation. Approximately 120 mg of lignin was dissolved in 500 μL of DMSO-*d*_6_, 60 μL of a relaxation agent (chromium(III) acetylacetonate), and 40 μL of internal standard (1,3,5 trioxane). The total concentration of lignin was 20% (w/v). The integration of the carbon moieties was based on the aromatic region (163–102 ppm), which was used as the reference of 6.12 carbon atoms assuming that it contains six aromatic carbon atoms with 0.12 of vinylic carbon atom. Therefore, the results are reported as aromatics per C_9_ lignin.^82,83^ Quantitative and qualitative ^13^C NMR spectra were obtained using a Bruker NEO 500 MHz spectrometer with respect to ^1^H, and it was equipped with a nitrogen-cooled cryoprobe. A total of 28,000 scans were acquired at 25 °C with a relaxation delay of 2 s.^84,85^

#### Fourier Transform Infrared Spectroscopy

Fourier transform infrared (FTIR) spectroscopy was carried out using a Thermo Scientific Nicolet 870 spectrometer. Predried potassium bromide (200 mg) and 1 mg of predried lignin were ground and pressed into a disc of 13 mm diameter where the thickness was approximately 0.6 mm. The samples were characterized in transmission mode where FTIR spectra were acquired using an average of 100 scans at a resolution of 4 cm^–1^. Omnic 8.1 software was used to analyze the spectra.

The condensation index of^18,86^ the lignin was calculated from the spectra using eq 2:
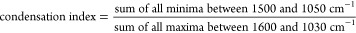
2

#### Differential Scanning Calorimetry

Differential scanning calorimetry-based analyses of the lignins were performed on a DSC-1 (Mettler Toledo Ltd., UK). Approximately 5.0 mg of the lignin samples (ARL, predried of AcSL and AcIL) was placed in a 40 μL aluminum pan and crimped with a prepierced aluminum lid. In the first scan, the sample was heated from 25 to 250 °C at 10 K·min^–1^and held isothermally for 3 min. A nitrogen atmosphere with a flow rate of 20 mL·min^–1^ was maintained throughout the experiment. The sample was cooled to 25 °C at 10 K·min^–1^ and held for 3 min. Two further sequential scans were performed as described above.

#### Thermo-Gravimetric Analysis

Thermo-gravimetric analysis (TGA) was performed using a Netzsch STA 449C (Germany) instrument. Approximately 10 mg of lignin was used in each experiment. TGA data were acquired as the sample was ramped from 25 to 900 °C at 10 K·min^–1^ in an argon atmosphere where the flow rate was 10 mL·min^–1^.

### Electro-Spinning of AcSL

#### Preparation of AcSL Solutions for Electro-Spinning

A 2:1 volume-to-volume ratio of acetone to DMSO, respectively, was used to dissolve the AcSL under reflux at ambient temperature. Five lignin concentrations corresponding to 45, 48, 53, and 58 wt % were made where the dissolution was carried out for 5 h at room temperature. These concentrations were selected because they formed the basis of a Taguchi-type experimental matrix where the requirement was to derive the processing parameters to obtain bead-free and unfused fibers. Dry nitrogen gas was bubbled into the solution at 20 mL·min^–1^ to create an inert blanket over the solution. A magnetic stirrer was also used to agitate the solution. The solutions were stored in an air-tight container until required.

#### Viscosity of the AcSL Solutions

The viscosity of the lignin solutions was determined using a parallel-plate rheometer (Discovery Hybrid Rheometer, model HR-1, UK). Forty millimeter diameter parallel-plates were employed in this study. The viscosity was measured by subjecting the sample to shear rates in the range 0.1 to 100 s^–1^ at 30 °C. A solvent trap was used to minimize the evaporation of acetone during the experiment.

#### Electrical Conductivity of AcSL Solutions

The electrical conductivity of the lignin solutions was measured using a Jenway 4510 Conductivity Meter (UK). Prior to undertaking these measurements, the equipment was calibrated at 25 °C using a standard sodium chloride solution (HI7033, Hanna instruments). The measurements were repeated three times where the temperature was maintained at 25 °C using recirculating water from a temperature-controlled water bath (Grant Instruments GD100, UK).

#### Electro-Spinning Rig

**Figure 1:**
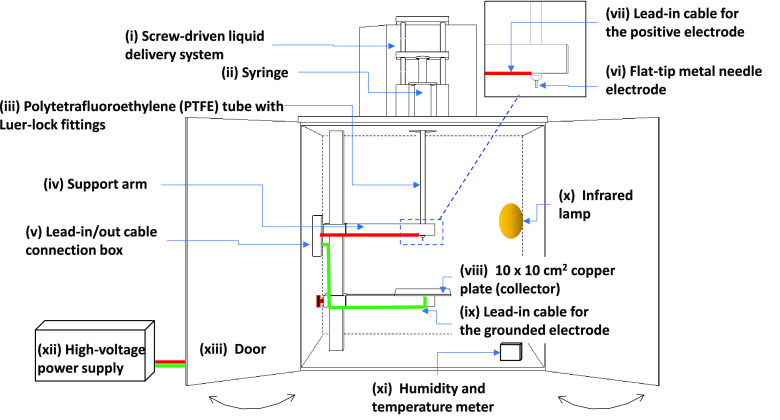
Schematic illustration of the experimental setup for electro-spinning.

A schematic illustration of the custom-designed electro-spinning unit is shown in Figure 1. The chamber and doors were constructed from a 10 mm thick polymethylmethacrylate sheet. A flat-tip metal needle (part number AD 725050, Adhesive Dispensing Ltd., UK) with an inner bore diameter of 0.254 mm was attached to a polytetrafluoroethylene tube with a bore diameter of 3 mm and secured to a syringe with a plunger (Masterflex transfer tubing, Cole-Parmer, UK) via Luer-lock connectors. The syringe assembly was attached to a precision liquid dispensing unit (model 941–371-1003, World Precision Instruments, UK), and the needle in turn was attached to a high-voltage power supply (Variable High-Voltage DC Power Supply, 73,030, Genvolt Ltd., UK) with a positive output polarity. A copper plate of dimensions 10 × 10 cm^2^ served as the grounded electrode. A piece of aluminum foil, with dimensions of 14 × 14 cm^2^, was secured on to the copper electrode, and the electro-spun fibers were deposited on it in a random fashion. The optimum processing parameters for electro-spinning were as follows. The working distance was set at 120 mm, and the applied voltage was 12 kV. The polymer dispensing rate depended on the viscosity of the solution, and the operating range was 0.1 μL·min^–1^.

**Figure 2:**
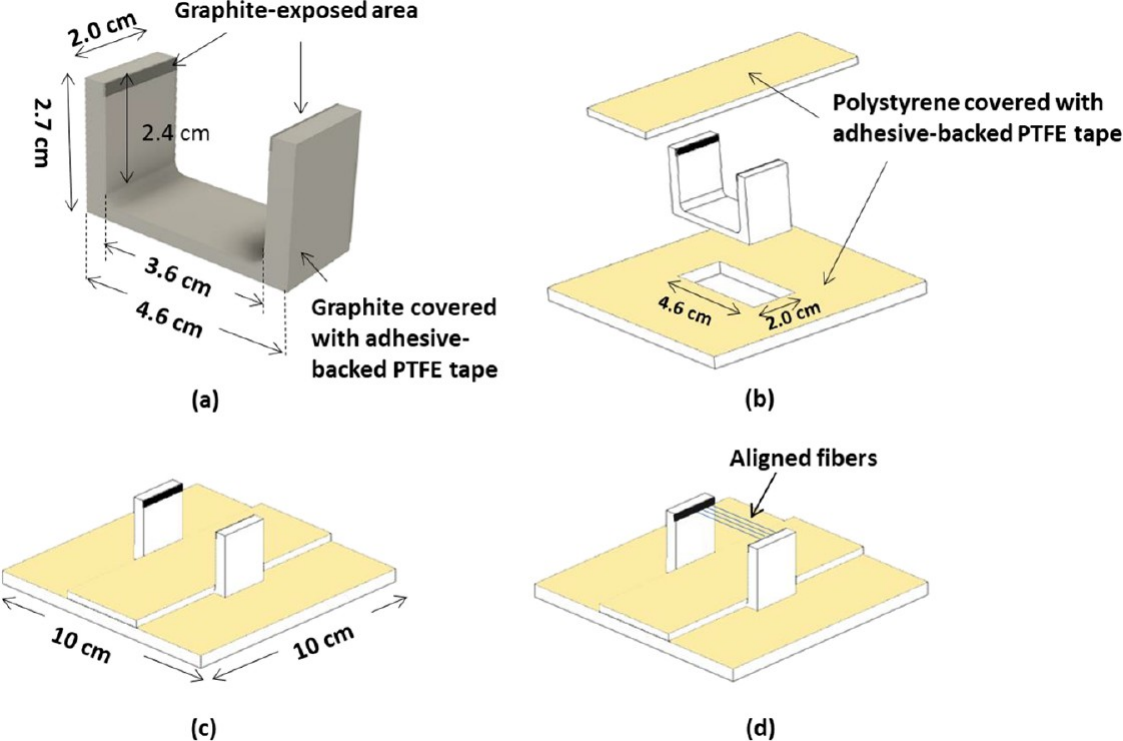
Schematic illustration of the graphite parallel electrodes for producing highly aligned electro-spun lignin fibers: (a) U-shaped graphite electrode, (b) components of the modified U-shaped graphite electrode and insulating 5 mm thick polystyrene sheet that was covered with adhesive-backed PTFE tape, (c) assembled electrode—placed on grounded copper electrode, and (d) orientation of the aligned electro-spun lignin fibers.

With regard to the production of aligned nanofibers, Figure 2a–d shows a schematic illustration of the: (a) U-shaped graphite electrode, (b) PTFE-coated polystyrene covers, (c) assembled fixture, and (d) fiber-alignment direction during electro-spinning. The graphite U-fixture was covered with an adhesive-backed PTFE tape except along the top inner edges and bottom-face. This assemble was placed on the grounded copper electrode. The two exposed top edges of the U-fixture acted as a pair of parallel electrodes^87,88^ where the electro-spun fibers were deposited perpendicular to the edges in an aligned manner. The thicknesses of the clear-polystyrene sheet and brown adhesive-backed PTFE tape (Part No. 5490 brown 50MMx33M, RS Pro, UK) were 5 and 0.09 mm, respectively. The distance between the tip of the needle to the top of the uncovered section of the grounded graphite electrode was 12 cm. During electro-spinning, the temperature and relative humidity in the electro-spinner chamber were maintained in the range 30–35 °C and 30%, respectively.

### Evaporating the Solvent from the AcSL Fibers

Since the solvent loading in the lignin solutions ranged from 42 to 55 weight percent, it was necessary to identify the optimum parameters for evaporating the solvent without causing the individual filaments to fuse. The treatments investigated were heating in: (i) an air-circulating oven, (ii) an oven with nitrogen gas flow, and (iii) a vacuum oven. In the first two heating cases, the electro-spun fibers were placed in an oven where the desired gas flow was achieved via the inlet and outlet gas ports, and this was maintained at 50 mL·min^–1^. Figure S1a (see the Supplementary Material) illustrates the experiment set up for heat treating the electro-spun AcSL fibers in an air-circulating oven or where a nitrogen gas flow was maintained. Dried-air (UN 1002, BOC, UK) and oxygen-free nitrogen (99.999% nitrogen, BOC, UK) were used. The drying regimes investigated were 100, 140, 180, or 200 °C for 6 h at each temperature.

With reference to heating the electro-spun fibers under vacuum, a schematic illustration of the vacuum oven and liquid nitrogen trap (to trap any volatile components) is shown in Figure S1b. To prevent atmospheric moisture from being introduced to the vacuum-dried fibers, the gas inlet port was fitted with a plastic bottle that was filled with silica gel before the inlet was opened to equilibrate the pressure. The dried fibers were sealed in plastic boxes and stored in a desiccator until required. With regard to the thermal treatments of the aligned fibers, the graphite fixture, with the fibers, illustrated in Figure 2d, was transferred to the vacuum oven to remove the solvent.

After drying the electro-spun lignin fibers as described above, they were transferred to a tube furnace (Pyro Therm Furnaces, UK) for oxidization and carbonization. A sacrificial alumina tube (ALM4638, Almath Crucibles Ltd., UK) with inner and outer diameters of 38 and 46 mm, respectively, and length of 1500 mm was inserted into the tube furnace. A schematic illustration of the experimental set up is shown in Figure S2. The electro-spun lignin fibers were heated in a ramp-and-hold sequence from room temperature to 100, 150, and 250 °C at 0.5 K·min^–1^ with a dwell of 1 h at each temperature. Air flow in the tube furnace was enabled using a compressed air cylinder where the gas flow was maintained at 50 mL·min^–1^. Prior to conducting these experiments, the tube furnace was calibrated to establish the temperature gradient from the center of the tube to the ends. After oxidative thermo-stabilization, carbonization was conducted under a constant nitrogen gas flow of 50 mL·min^–1^. The oxidized lignin fibers were heated from their respective thermo-oxidation temperature to 1000, 1200, and 1500 °C at 5 K·min^–1^ with a dwell of 1 h before cooling naturally to room temperature.

### Characterizations of AcSL Fibers, Heated, and Carbonized AcSL Fibers

#### Scanning Electron Microscopy

A scanning electron microscope (SEM, Hitachi 3030, Japan) was used to characterize the morphology of the electro-spun fibers. The samples were gold-coated prior to inspection. The SEM was operated using an accelerating voltage of 15 kV. Fiber diameter distribution was measured using ImageJ software. A comparison of fiber diameters was conducted by ANOVA using Minitab17 at a significance level of 0.05.

#### Raman Spectroscopy

A Raman spectrometer (Renishaw RE-04, UK) equipped with a 488 nm laser diode was used to investigate the graphitic structure of carbonized lignin fibers. Spectra were obtained through a 50× microscope objective over 100 scans^1^. The Raman spectra were curve-fitted using a Gaussian/Lorentzian distribution. The intensity ratio of the D and G bands (*I*_D_/*I*_G_) was used to describe the degree of graphitization and to calculate the crystallite size using eq 3:
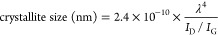
3where λ is the output wavelength of the laser in nm.

## Results and Discussion

### Characterizations of ARL, AcSL, and AcIL

The purity, homogeneity, degree of branching, and molecular weight distribution of lignin precursors are some of the key parameters that can influence the quality of carbonized fibers. Table 2 shows the ash content, molecular weight distribution, and polydispersity index for ARL, AcSL, and AcIL and acid-washed lignin. The ash content in the ARL was 1.20 ± 0.02%, and this is similar to that reported in the literature for BioChoice lignin.^17,18^ Acid-washing is a conventional technique that is used to removed inorganics in lignin.^33,34,46,64,66^ In the current work, ARL was treated with a 37% aqueous solution of hydrochloric acid at pH 2, and the ash content was 0.35 ± 0.02%. After fractionation, the ash contents in the AcSL and AcIL were 0.06 ± 0.02 and 2.4 ± 0.03%, respectively. The lowest ash content was found in AcSL, and its fractionation yield was 57.4 ± 0.6%. The inorganic content in lignin is a concern as it can result in the formation of defect in the fibers and thus degrade the desired mechanical properties.^10^ Moreover, elementals such as sodium and potassium are known to act as catalysts for the chemical cracking of lignin.^89−92^

**Table 2:** Ash Content, Molecular Weight Distribution, and Polydispersity Index for ARL, AcSL, and AcIL and ARL Washed with Acidified Water at pH 2

sample	ash content (%)	*M*_w_ (g·mol^–1^)	*M_n_* (g·mol^–1^)	PDI
ARL	1.20 ± 0.02	6000 ± 283	2700 ± 141	2.22 ± 0.22
AcSL	0.06 ± 0.02	4250 ± 71	2450 ± 71	1.73 ± 0.07
AcIL	2.4 ± 0.03	9600 ± 566	3400 ± 141	2.82 ± 0.28
ARL washed with acidified-water at pH 2	0.35 ± 0.02	N/A		

The weight average molecular weight (*M*_w_) of the AcSL was 4250 ± 71 g·mol^–1^, and this is 29% lower than that of the ARL. The molecular weight distribution traces for ARL, AcSL, and AcIL are shown in Figure S3. The polydispersity index (PDI) of ARL was 2.22 ± 0.22, and it was reduced to 1.73 ± 0.07 in the AcSL fraction. This indicates an improvement in the homogeneity of lignin when compared to its as-received state. The PDIs of ARL and AcSL were lower than that of AcIL (2.82 ± 0.28). The *M*_w_ and PDI of the soluble and insoluble fractions show a similar trend to those reported in literature.^93−96^ In the case of synthetic polymers, a PDI in the range 1.5–2.0 is reported to favor fiber formation.^50^

^13^C NMR spectroscopy was used to identify and quantify certain carbon lignin moieties in ARL, AcSL, and AcIL.^97^ The ^13^C NMR spectra showed better signal resolution upon the derivatization (acetylation) of lignin. The ^13^C NMR spectra for ARL and its acetylated counterpart (Ace-ARL) are shown in Figure S4. The assignments with integration of certain carbon moieties in the structure of ARL including soluble and insoluble lignin fractions are shown in Table S1. The chemical shift at 92 ppm corresponds to the internal standard (1,3,5-trioxane), which was used for the quantification of carbon moieties in the lignin. There are signals with weak intensities between 102 and 98 ppm, and this could be indicative of the presence of carbohydrates. This correlates with the composition of BioChoice lignin where the presence of small impurities of sugars has been observed.^17,98^ The chemical shifts between 102 and 160 ppm are attributed to aromatic carbon moieties within the lignin structure. The characteristic peaks in acetylated lignin trace from 165 to 172 ppm are assigned to hydroxyl groups in lignin. The most noticeable difference is in the ratio of hydroxyl groups in the phenolic and aliphatic region. This ratio correlates with the ^31^P NMR results reported previously by the authors for lignin, which showed a higher ratio of phenolic-to-aliphatic hydroxyl groups for AcSL.^97^

FTIR spectra for the ARL and AcSL fractions are shown in Figure S5, and the absorbance band assignments are compiled in Table S2. The characteristic O–H stretching (3400–3410 cm^–1^), C–H stretching at 2930 and 2830 cm^–1^, and aromatic skeleton vibrations at 1590 and 1510 cm^–1^ can be observed in the spectra. Softwood lignin, which has a higher guaiacyl content shows the presence of C–O and C=O stretching at 1264 cm^–1^. The two peaks at 855 and 815 cm^–1^ are assigned to out-of-plane C–H deformation in the G-units of lignin. FTIR spectra can be used to determine the condensation index (CI);^18,86^ this relates to the C–C linkages in lignin. The CI for AcSL was reduced to 0.66 from 0.72 for the ARL, while it increased to 0.75 for the AcIL fraction. A higher IC is reported for softwood lignin^18^ when compared to herbaceous and hardwood lignin. This is because softwood is composed of guaiacyl units, which allow for extensive C–C bonding at the 5–5′ position.

With regard to the properties of AcSL, fractionation using acetone is a simple and one-step procedure for the pretreatment of softwood Kraft lignin prior to electro-spinning. It enables the removal of inorganic impurities, reduces the PDI and the condensation index, and enhances the ratio of the phenolic-to-aliphatic hydroxyl group.

The thermal properties of AcsL and AcIL were investigated and compared with the ARL using DSC and TGA. The experimentally derived *T*_g_ data from the DSC analyses were used to define the temperature regimes for the subsequent drying and oxidative thermo-stabilization of the electro-spun fibers with a view to prevent fiber fusion. A summary of the data is presented in Table 3. Selected DSC thermograms corresponding to three consecutive heating scans for the ARL, AcsL, and AcIL are presented in Figure S6a–c. In the first heating scan, broad endothermic peaks were observed for the ARL, AcSL, and AcIL; the endothermic peaks for the three lignins were at 87.0, 76.8, and 83.5 °C, respectively. These endotherms are likely to be caused by the evaporation of water and low-molecular-weight volatile components in the lignin. In the first heating scan, the *T*_g_ values of the ARL, AcSL, and AcIL samples were 150, 127, and 178 °C, respectively. In the second heating scan, the previously mentioned endotherms were not observed but instead a single *T*_g_ was observed. The ARL showed a *T*_g_ at 163 °C, and those for the AcSL and AcIL were 146 and 189 °C, respectively. The lower *T*_g_ for the AcSL may be attributed to the lower molecular weight distribution and PDI as shown in Table 2. In the third heating scan, the *T*_g_ was observed to increase by approximately 8–9 °C when compared to the second heating scan (see Table 3). The observed increase in the *T*_g_ after each heating scan may be attributed to the fact that the lignin was heated to 250 °C in each experiment thus leading to cross-linking.^99,100^

**Table 3:** DSC and TGA Data for ARL, AcSL, and AcIL

	DSC data					TGA data		
	1st scan			2nd scan	3rd scan			
samples	endothermic peak (°C)	enthalpy (J·g^–1^)	*T*_g_ (°C)	*T*_g_ (°C)	*T*_g_ (°C)	mass at 900 °C (%)	*T*_DTG,max_ (°C)	maximum mass-loss rate (%/°C)
ARL	87.0 ± 0.9	65.7 ± 7.3	150.2 ± 6.8	163.0 ± 1.5	172.2 ± 0.4	45.0 ± 1.2	391.5 ± 10.9	2.6 ± 0.1
AcSL	76.8 ± 0.7	41.4 ± 6.6	127.5 ± 1.6	145.8 ± 0.3	151.8 ± 1.9	36.5 ± 1.4	393.6 ± 10.8	3.1 ± 0.2
AcIL	83.5 ± 0.5	96.9 ± 6.9	178.0 ± 0.5	189.1 ± 0.4	196.6 ± 0.9	41.0 ± 3.7	387.8 ± 5.8	2.9 ± 0.1

The TGA mass-loss and first derivative of the mass-loss with respect to temperature (DTG) traces for the three lignin samples are presented in Figure S7a,b, and the results are compiled in Table 3. The char contents at 900 °C obtained from ARL, AcSL, and AcIL were 45.0, 36.5, and 41.0%, respectively. With reference to Figure S7, in stage-1, the DTG traces for the three lignin samples show a small broad peak below 150 °C; this is attributed to the evaporation of moisture and low-molecular-weight components.^101−103^ In stage-2, a small mass-loss peak is observed between 161 and 192 °C. However, the ARL and AcIL exhibit mass-loss onset peaks at 287 and 282 °C, respectively. This may be due to the degradation of lignin-carbohydrate complexes with larger molecular weight components when compared to AcSL.^17,104^ The rate of mass-loss for the AcIL in stage-2 is lower than that compared to ARL and AcSL. This is probably due to the chemical composition of this fraction and its molecular weight distribution as seen in Figure S3. The presence of a shoulder is evident for the ARL between 278 and 285 °C, but its prominence is significantly lower for the AcSL and ASL. The reason for the presence of this shoulder is not known at present but since the sample with the prominent shoulder (ARL) shows the highest mass-retention at 900 °C, investigating the mechanistic reasons for this may enable the production of a higher char content at and above 900 °C. Furthermore, there is a noticeable faster rate of mass-loss after the shoulder observed in stage-2 (see Figure S7). In stage-3, the observed derivative of the mass-loss data at 310 and 600 °C is said to be due to the degradation of lignin.^97,103,105^ The mass-loss rates for the ARL, AcSL, and AcIL are 2.6, 3.1, and 2.9%/°C. This mass-loss rate seems to be related to the molecular weight distribution for the AcSL and this trend correlates well with results reported in the literature.^19,93,106^ The data shown in Figure S7 suggest that TGA data can be used to screen naturally occurring materials to assess their potential as precursors for the production of carbonized fibers.

With reference to Tables 2 and 3, AcSL lignin was chosen as the material for the electro-spinning experiments for the following reasons. (i) The AcSL had the lowest ash content (0.06%) when compared to the ARL (1.2%) and AcIL (2.4%). It is known that impurities can compromise the mechanical properties of the carbonized fibers. (ii) Although AcSL had the lowest molecular weight distribution in comparison to the ARL and AcIL, it will be shown in a subsequent section that it was sufficient to enable it to be electro-spun at the appropriate concentration and processing conditions. (iii) AcSL registered the lowest enthalpic peak area during the first DSC scan, and therefore by implication, the lowest evolution of volatiles during heat treatment. (iv) The *T*_g_ for AcSL was 127.5 °C (first scan), and this was the lowest when compared to ARL (150.2 °C) and AcIL (178 °C). This meant that it could be heated to just above the *T*_g_, in a vacuum oven, to evaporate the solvent. (v) The second and third DSC scans demonstrated that the *T*_g_ increased with sequential heating to 250 °C and this is attributed to cross-linking. This meant that the fibers could be heat-treated past the *T*_g_ while retaining their circular cross-section. Furthermore, the thermograms did not show any obvious indication of thermal degradation. (vi) The mass remaining after heating the lignin to 900 °C in an inert atmosphere for the AcSL was 36.5%. This was deemed sufficient to demonstrate the electro-spinning of unblended lignin, and to optimize the processing parameters. (viii) The AcSL fraction is fully soluble in acetone at room temperature, and therefore, no further treatments, blending, or functionalization was required to electro-spin it.

### Electro-Spinning of Acetone-Soluble Lignin

Although AcSL is soluble in acetone, the resulting lignin solution could not be electro-spun for extended periods due to the clogging of the spinneret. This problem is attributed to the low boiling point of acetone (56 °C), and it was overcome by using a binary combination of acetone and DMSO. AcSL is soluble in DMSO, and its boiling point is 189 °C. With reference to the use of the binary solvent with significantly different boiling points, the hypothesis was that the evaporation of acetone from the outer circumference of the electro-spun fiber would create a “skin” hence enabling the production of unfused fibers. To determine the optimal binary solvent concentration for the acetone-to-DMSO ratio, a series of experiments were performed with varying volume ratios of the solvents corresponding to 0 (single solvent (acetone)), 4:1, 3:1, and 2:1. The total concentration of AcSL was kept constant at 53 wt %. The observations made during the production of the fibers are compiled in Table S3. It was concluded that a volume ratio of 2:1 of acetone:lignin was the optimum solvent ratio to produce continuous electro-spun fibers without the occurrence of clogging at the tip of the needle. In addition, AcSL concentrations of 45, 48, 53, and 58 wt % in a binary solvent of acetone and DMSO (2:1 volume ratio) were also prepared to investigate the morphology of the electro-spun fibers.

Typical traces for the viscosity versus shear rate for AcSL solutions at specified concentrations are shown in Figure S8. The traces show shear-thinning behavior, and the extrapolated viscosities at zero shear rate were 0.23, 0.31, 0.43, and 0.63 Pa·s. The electrical conductivity of the lignin solutions ranged from 2.29 to 2.41 μS·cm^–1^, and it was observed to increase with increasing lignin concentration.

**Figure 3:**
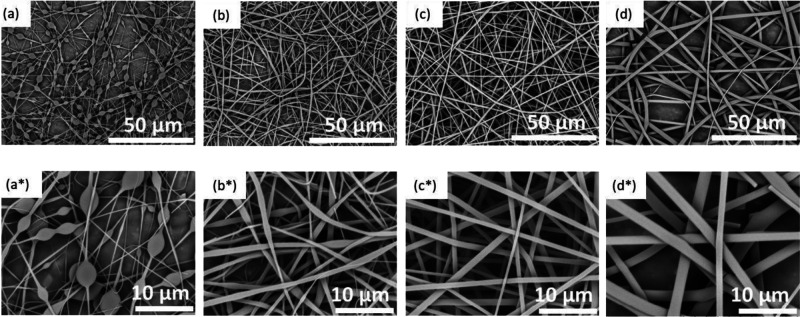
(a–d) SEM micrographs showing the morphology of the randomly oriented electro-spun AcSL fibers at lignin concentrations of (a) 45, (b) 48, (c) 53, and (d) 58 wt %, respectively. The top (a–d) and bottom (a*–d*) rows show micrographs at 1500× and 5000× magnification, respectively.

Figure 3a–d shows micrographs for the as-spun fibers obtained from lignin solution concentrations corresponding to 45, 48, 53, and 58 wt %. The micrographs have been coded and paired with the low and higher magnification micrographs at the top and bottom rows, respectively; in other words, a series of micrographs coded as “a–d” and “a*–d*” represent low- and high-magnification images, respectively. At a lignin concentration of 45 wt %, beaded fibers were observed (see Figure 3a,a*). By increasing the concentration of lignin to 48 wt %, the beads on the electro-spun fibers were eliminated; however, the fibers resembled ribbons (Figure 3b,b*). When the concentration of lignin was increased to 53 wt %, bead-free electro-spun fibers were produced (Figure 3c,c*) and continuous electro-spinning was possible without the spinneret clogging. However, increasing the lignin concentration to 58 wt % caused an increase in the presence of ribbon-like fibers (Figure 3d,d*) and their relative widths increased.

**Figure 4:**
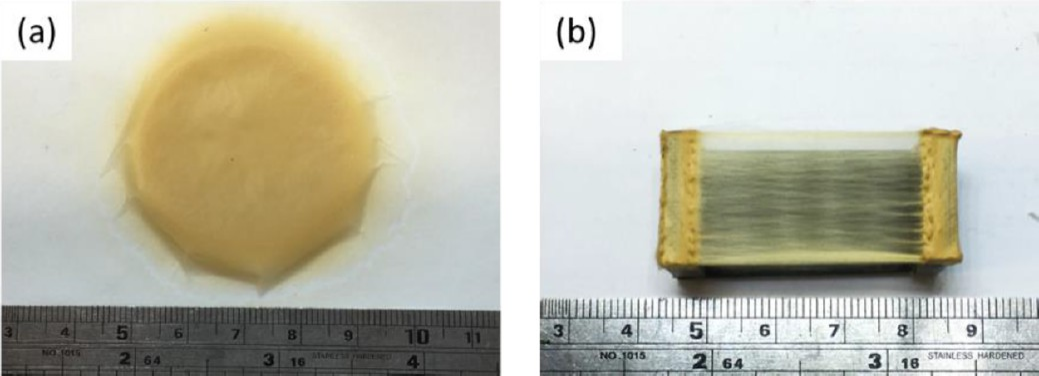
Appearance of the deposition area for the as-spun lignin fibers using a 53 wt % solution in a 2:1 of acetone-to-DMSO: (a) randomly oriented AcSL fiber mat collected by the flat-plate electrode and (b) highly aligned fibers collected by modified parallel graphite electrodes.

In the above-mentioned experiments, the 53 wt % AcSL in a 2:1 volume ratio of acetone/DMSO enabled continuous electro-spinning where the electro-spun fibers exhibited a circular cross-section and the fractured surfaces were defect-free. After 5 min of electro-spinning, the diameter of the deposition area for the randomly orientated electro-spun mat was approximately 5 cm; this is shown in Figure 4a. The macroscopic appearance of the aligned fibers that were deposited on the graphite parallel electrodes is shown in Figure 4b. The fibers are aligned perpendicular to the two parallel electrodes that were separated by 3.6 cm.

**Figure 5:**
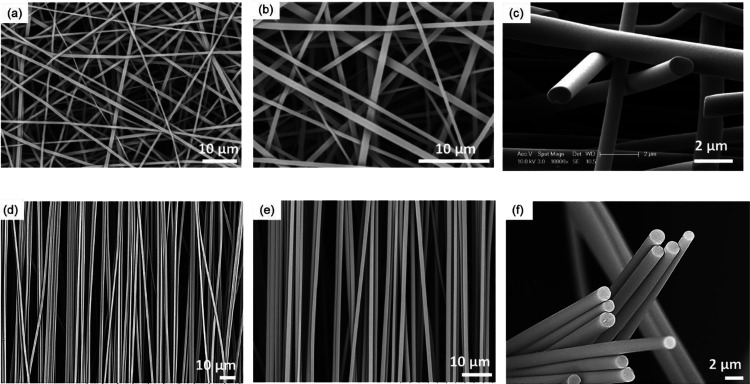
SEM micrographs showing aligned and randomly oriented as-spun AcSL fibers: (a–c) randomly oriented fibers; and (d–f) aligned fibers at different magnifications. The fibers were electro-spun from a 53 wt % lignin solution in a 2:1 ratio of acetone-to-DMSO.

SEM micrographs of as-spun randomly oriented and aligned AcSL fibers are shown in Figure 5a–c,d–f, respectively, where the surface is observed to be smooth and without any surface defects. The transverse cross-sections shown in Figure 5c,f demonstrate that the fibers are void-free with a near circular cross-section. The diameters of the randomly oriented and aligned fibers were measured using ImageJ software where six individual SEM micrographs each were taken from six randomly selected areas. Three hundred individual fibers were measured in each case. The diameter of the randomly oriented electro-spun AcSL was found to be in the range 0.5–1.8 μm with an average diameter of 1.16 ± 0.21 μm. The diameter range for the aligned fibers was 0.8–2.4 μm with an average of 1.48 ± 0.23 μm. Histogram plots for the diameters, with overlaid normal distributions, for the randomly oriented and aligned electro-spun AcSL fibers are presented in Figures S9a and S10a, respectively. The fibers diameter distribution for the randomly oriented electro-spun AcSL fibers were used to investigate the changes in diameter after subsequent thermal treatments.

**Figure 6:**
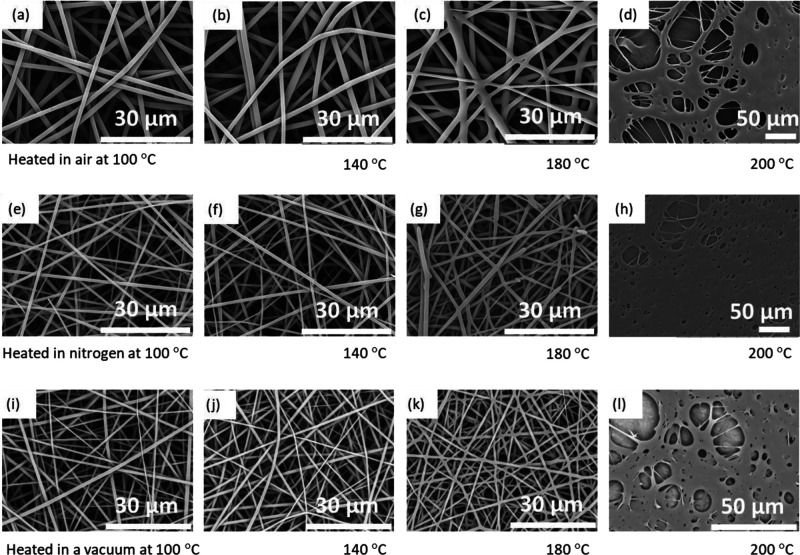
Fiber morphology as a function of heat treatment temperatures and environment. The fibers were electro-spun from a 53 wt % lignin solution in a 2:1 ratio of acetone-to-DMSO. Morphology of the fibers that were heat treated at 100, 140, 180, and 200 °C in: air (a–d), nitrogen (e–h), and a vacuum oven (i–l), respectively.

Prior to thermo-stabilization, it was necessary to heat-treat the electro-spun fibers to remove the solvents. This was done to prevent the fusion of fibers during thermo-stabilization and carbonization. The morphologies of the electro-spun fibers that were heated at 100, 140, 180, and 200 °C for 6 h, in air, nitrogen, and in a vacuum oven are shown in Figure 6. Evidence for fiber fusion was not observed when the electro-spun fibers were heated from 100 to 140 °C, in air, nitrogen, or a vacuum oven (see Figure 6a,b,e,f,I,j). However, as seen in Figure 6c,g,k, the fibers were seen to be fused at the fiber cross-over regions at approximately 180 °C for the three heat treatment conditions. At 200 °C, extensive fiber fusion is observed as shown in Figure 6d,h,i. Since the boiling point of DMSO is 189 °C and the *T*_g_ of lignin from the first DSC scan is 127 °C, evaporating it at 140 °C would have been time-consuming. Hence, the solvent in the electro-spun fibers was removed by heating them at 140 °C in a vacuum oven for 6 h.

The transitions in the morphology of the electro-spun AcSL fibers after heat treatment in a vacuum oven at 140 °C for 6 h and those that were thermo-stabilized in air at 250 °C for 1 h are presented in Figure S11. The diameter distributions for these AcSL fibers are shown in Figure S9a–c. Fiber diameters for the above-mentioned heat treatments range from 0.3 to 1.8 μm, and the average diameter are 1.16 ± 0.20, 1.16 ± 0.22, and 1.14 ± 0.21 μm, respectively.

**Figure 7:**
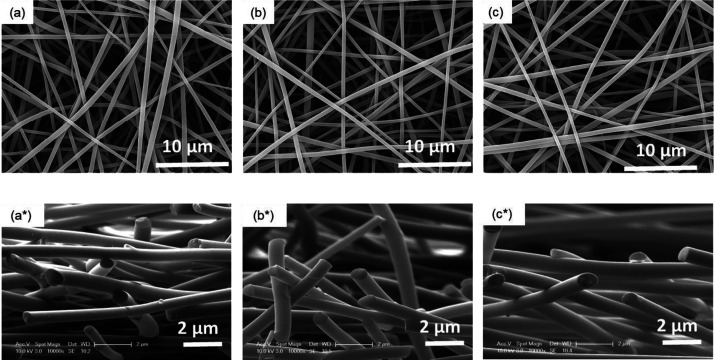
(a–c) SEM micrographs for AcSL fibers after carbonization at 1000, 1200, and 1500 °C, respectively. The top row (a–c) shows the surface morphology, and the bottom row (a*–c*) represents the transverse cross-sections.

After thermo-stabilization in air, the AcSL fibers were carbonized at 1000, 1200, and 1500 °C under a nitrogen atmosphere. SEM micrographs for the surface morphology and cross-section of carbonized AcSL lignin fibers are shown in Figure 7. The samples are seen to have a smooth surface without any evidence for fiber fusion or voids. The transverse micrographs of the electro-spun fibers illustrate that they have a circular cross-section and this makes them suitable for subsequent use as reinforcements in the production of composites. A narrower fiber diameter distribution is observed for the carbonized fibers as shown in Figure S9d–f when compared to those that were thermo-stabilized in air (Figure S9c). The diameters of fibers thermo-stabilized in air at 250 °C and carbonized in nitrogen at 1000 °C were 1.14 ± 0.21 and 526 ± 148 nm, respectively. This reduction in the fiber diameter is expected due to thermally induced shrinkage and mass-loss during carbonization as evident in the data obtained from the TGA analysis. Increasing the carbonization temperature from 1000 to 1200 °C resulted in a small reduction in the fiber diameter to 503 ± 124 nm. A further increase in the carbonization temperature to 1500 °C did not have a noticeable influence on the fiber diameter. The average of diameter of the obtained fibers after carbonized at 1500 °C was 502 ± 125 nm. A similar trend was observed for the aligned AcSL fibers where, after carbonization at 1000, 1200, and 1500 °C, the diameters were 639 ± 94, 551 ± 74, and 547 ± 95 nm, respectively (Figure S12). Histogram plots with overlaid normal distributions for the measured fibers diameter are presented in Figure S10b–d.

**Figure 8:**
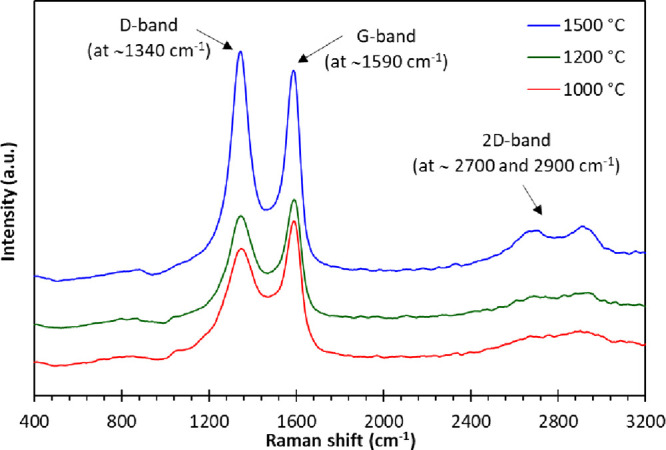
Raman spectra of carbonized electro-spun lignin fibers obtained after carbonization at 1000, 1200, and 1500 °C.

Raman spectroscopy was conducted to evaluate the graphitic structure of the AcSL fibers carbonized at 1000, 1200, and 1500 °C, and the spectra are presented in Figure 8. Two broad characteristic bands can be seen in the spectra: the D-band at 1340 cm^–1^ and the G-band at 1590 cm^–1^.^107−111^ The D-band represents the breathing mode of carbon atom in an aromatic ring, and this band indicates the presence of disordered carbon. The G-band on the other hand is attributed to in-plane stretching of sp^2^ carbon, and this is taken to represent an ordered carbon structure.^46,112−116^ The bands at approximate 2700 and 2900 cm^–1^ were noticeable as the carbonization temperature was increased from 1000 to 1500 °C. These bands are second-order resonances of the D-band, and they represented stacking in layered graphitic sheets.^117^

Gaussian curve fitting was carried out to analyze the band width and the corresponding area under the peaks. The peak intensity ratio of the D-band to the G-band (*I*_D_/*I*_G_) is used to describe the degree of graphitization, and the data are compiled in Table 4. With reference to Table 4, it is seen that the *I*_D_/*I*_G_ ratio increased from 0.82 ± 0.03 to 0.86 ± 0.02 and 1.00 ± 0.08 as the carbonization temperature was increased from 1000 to 1200 °C and 1500 °C, respectively. This observed trend is counter intuitive because in the case of PAN, the *I*_D_/*I*_G_ ratio decreases as a function of increasing carbonization and graphitization temperature. However, consensus has not been reached in the literature for lignin as some researchers have reported an increase^25,72,97,111,118^ and other a decrease^73^ in the *I*_D_/*I*_G_ ratio with increasing carbonization temperature. The degree of a disorder structure in lignin with increasing carbonization-carbonized lignin is ascribed to the irregular and complex structure of lignin.^118−120^ The crystallite size (*L*_a_) of the graphitized lignin was found to be 16.63, 15.84, and 13.67 nm for the fibers that were carbonized at 1000, 1200, and 1500 °C. A similar crystallite size range was reported for fibers produced via electro-spinning of lignin grafted with polyacrylonitrile.^73,121^

**Table 4:** Structural Parameters Obtained from Raman Spectroscopy for Lignin Fibers That Were Carbonized at 1000, 1200, and 1500 °C

carbonization temperatures	*I*_D_/*I*_G_	*L*_a_ (nm)	D-band FWHM (cm^–1^)	G-band FWHM (cm^–1^)
1000 °C	0.82 ± 0.03	16.63 ± 0.45	211.7 ± 1.0	103.1 ± 9.9
1200 °C	0.86 ± 0.08	15.84 ± 0.37	171.8. ± 2.1	101.0 ± 1.0
1500 °C	1.00 ± 0.02	13.67 ± 1.29	104.1 ± 0.8	83.5 ± 0.9

The full-width at half-maximum (FWHM) for the D and G-bands shown in Table 4 decreased with increasing carbonization temperatures. The FWHM for the G-band is seen to narrower when compared to the D-band. This implies that the development of an ordered graphitic structure increases with increasing carbonization temperatures.^25,73,118^

## Conclusions

In this work, the production of carbonized lignin nanofibers from 100% softwood Kraft lignin was demonstrated for the first time. It was shown that single-solvent fractionation using acetone can reduce the ash content and improve the polydispersity index of lignin; the acetone-soluble lignin fraction (AcSL) had an ash content of 0.06 wt %, a *M*_w_ of 4250 g·mol^–1^, and a PDI of 1.7. To enable electro-spinning, AcSL was dissolved in a binary solvent of acetone and dimethyl sulfoxide. The optimal solution concentration to produce fibers with a circular cross-section was 53 wt % in a 2:1 ratio of acetone: DMSO. A custom-made electro-spinning unit was used to produce random mat and uniaxially aligned fiber preforms. In the latter case, a custom-made graphite electrode was used as it enabled the electro-spun fibers to be heat treated (to remove the solvent), oxidized, and carbonized without having to remove the fibers from the rig.

Prior to oxidizing the fibers in air, the optimal heat treatment to remove the solvent without causing fiber fusion was vacuum drying at 140 °C for 6 h. Thermo-stabilization was carried out by heating the predried fibers from ambient to 250 °C at 0.5 K·min^–1^ in air. Carbonization was carried out in a nitrogen gas atmosphere where the fibers were heat treated to 1000, 1200, and 1500 °C, and the diameters were 639 ± 94, 551 ± 74, and 547 ± 95 nm, respectively. The carbonized fibers retained their circular cross-section, and they were not fused. SEM micrographs representing the transverse cross-section of carbonized fibers showed that the fibers were void-free. The protocol developed in this study can be used to produce carbonized lignin fibers that can be used in a plethora of applications including the production reinforcements for composites, filtration technologies, and mats for absorbing heavy metal ions.

## Supporting Information Available

Experimental setup for heat treating the electro-spun fibers in specified environments (Figure S1); illustration of the setup for oxidizing and carbonizing the lignin fibers (Figure S2); molecular weight distribution data for the lignins investigated (Figure S3); ^13^C NMR spectra for as-received lignin and acetylated as-received lignin (Figure S4); integration and assignment of ^13^C NMR for specified lignins (Table S1); FTIR spectral assignments (Figure S5); spectral absorption band assignments for the lignins investigated (Table S2); DSC thermograms for specified lignins (Figure S6); mass-loss versus temperature and derivative for the lignins (Figure S7); experiment detail, viscosity, and electrical conductivity of acetone-soluble lignin solutions and observations during electro-spinning (Table S3); relationship between the viscosity and shear rate (Figure S8); Fiber diameter distribution plots for randomly oriented acetone-soluble lignin fibers (Figure S9); fiber diameter distribution plots for aligned acetone-soluble lignin fibers with and without heat treatment (Figure S10); SEM micrographs for the acetone-soluble randomly oriented electro-spun lignin fibers with and without heat treatment (Figure S11); and SEM micrographs representing aligned lignin fibers that were carbonized (Figure S12) (PDF)
