# Supplementary material for: Single-Solvent Fractionation and Electro-Spinning Neat Softwood Kraft Lignin
Source: ACS Appl Bio Mater. 2023 Jul 31;6(8):3153–65. doi: 10.1021/acsabm.3c00278 (PMC10445268; doi:10.1021/acsabm.3c00278)
Supplement: Supplementary file 1 — mt3c00278_si_001.pdf [file mt3c00278_si_001.pdf]

Supporting Information

Single-Solvent Fractionation and Electro-spinning Neat Softwood Kraft Lignin

*Bongkot Hararak, Inam Khan, Gerard F Fernando**

Sensors and Composites Group, School of Metallurgy and Materials, The University of Birmingham, Edgbaston, Birmingham, B15 2TT, United Kingdom

Correspondence to: Gerard F Fernando (E-mail: g.fernando@bham.ac.uk)

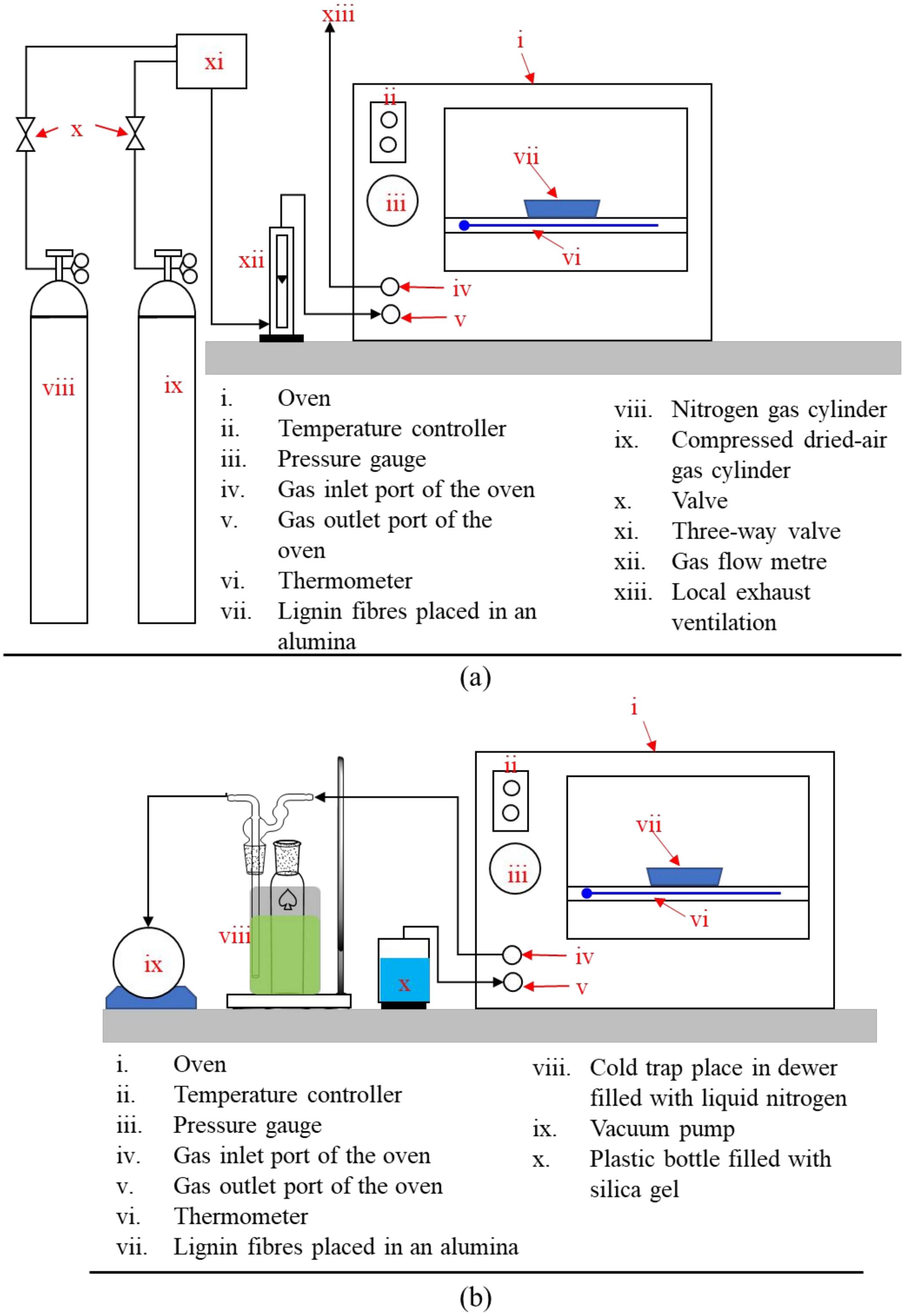

Figure S1. Schematic illustration of the experimental setup for heat treating the electro-spun acetone-soluble fibres under (a) a nitrogen or compressed air atmosphere and (b) vacuum.

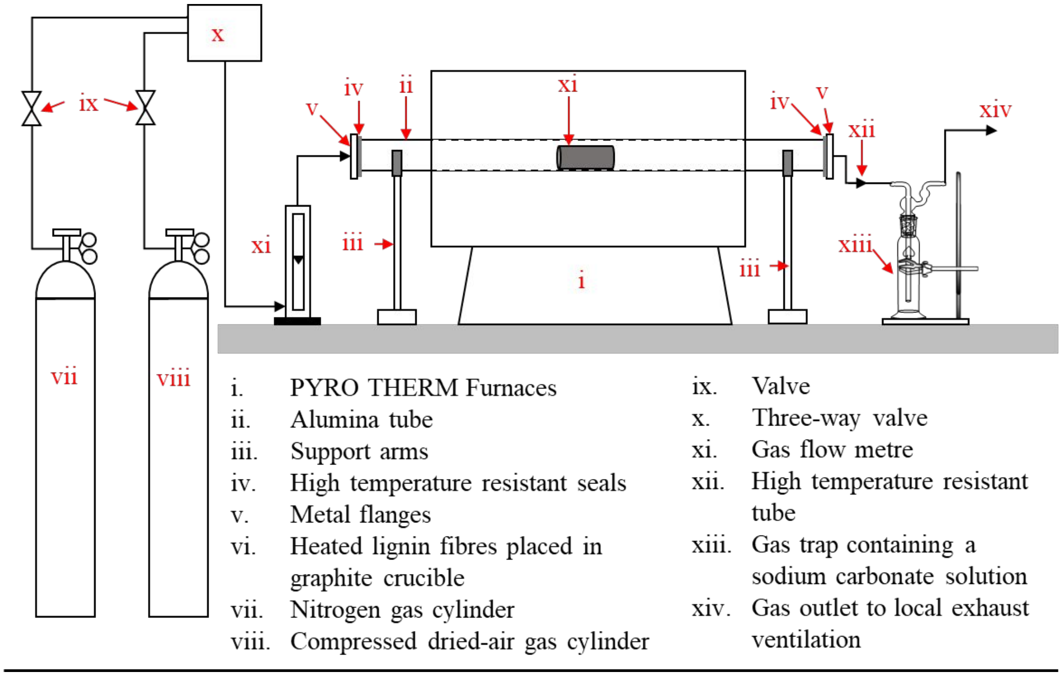

Figure S2. Schematic illustration of the experimental setup for thermo-stabilising and carbonising the electro-spun acetone-soluble lignin fibres.

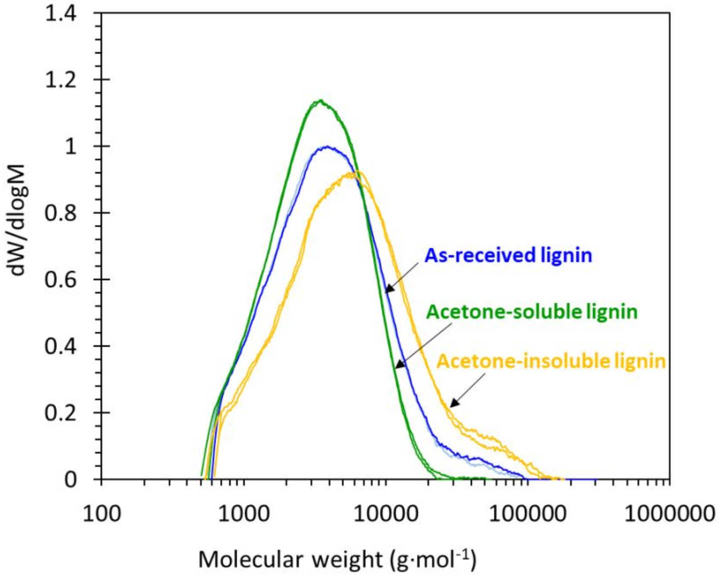

Figure S3. Molecular weight distribution data for the as-received, acetone-soluble and acetone-insoluble lignins.

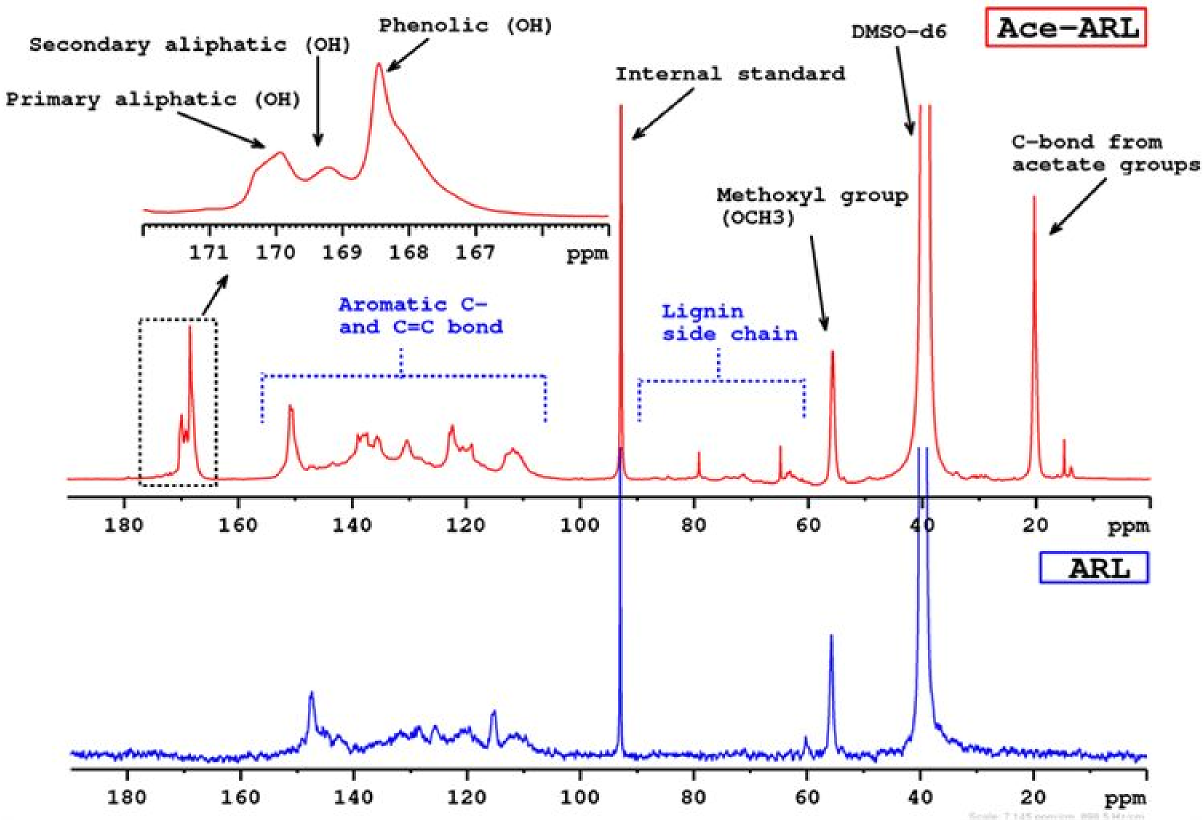

Figure S4. ^{13}C NMR spectra for as-received lignin and acetylated as-received lignin.

Table S1. Integration and assignment of ^{13}C NMR for acetylated as-received lignin, acetylated acetone-soluble and acetylated acetone-insoluble lignin.

Chemical Shift, δ (ppm)	Assignment	Number of carbon atoms per aromatic group		
		Acetylated as-received lignin	Acetylated acetone-soluble lignin	Acetylated acetone-insoluble lignin
171.8-165.6	Total OH groups	1.25	1.54	1.07
171.8-169.6	Primary Aliphatic	0.31	0.23	0.33
169.6-168.9	Secondary Aliphatic	0.19	0.14	0.19
168.9-165.6	Phenolic	0.75	0.80	0.55

163.0-102.0	Aromatic carbons	6.12	6.12	6.12
90.0-58.5	Aliphatic carbon side chain or C-O	0.46	0.97	0.29
58.0-54.0	Methoxyl (OMe) group	0.78	0.87	0.57

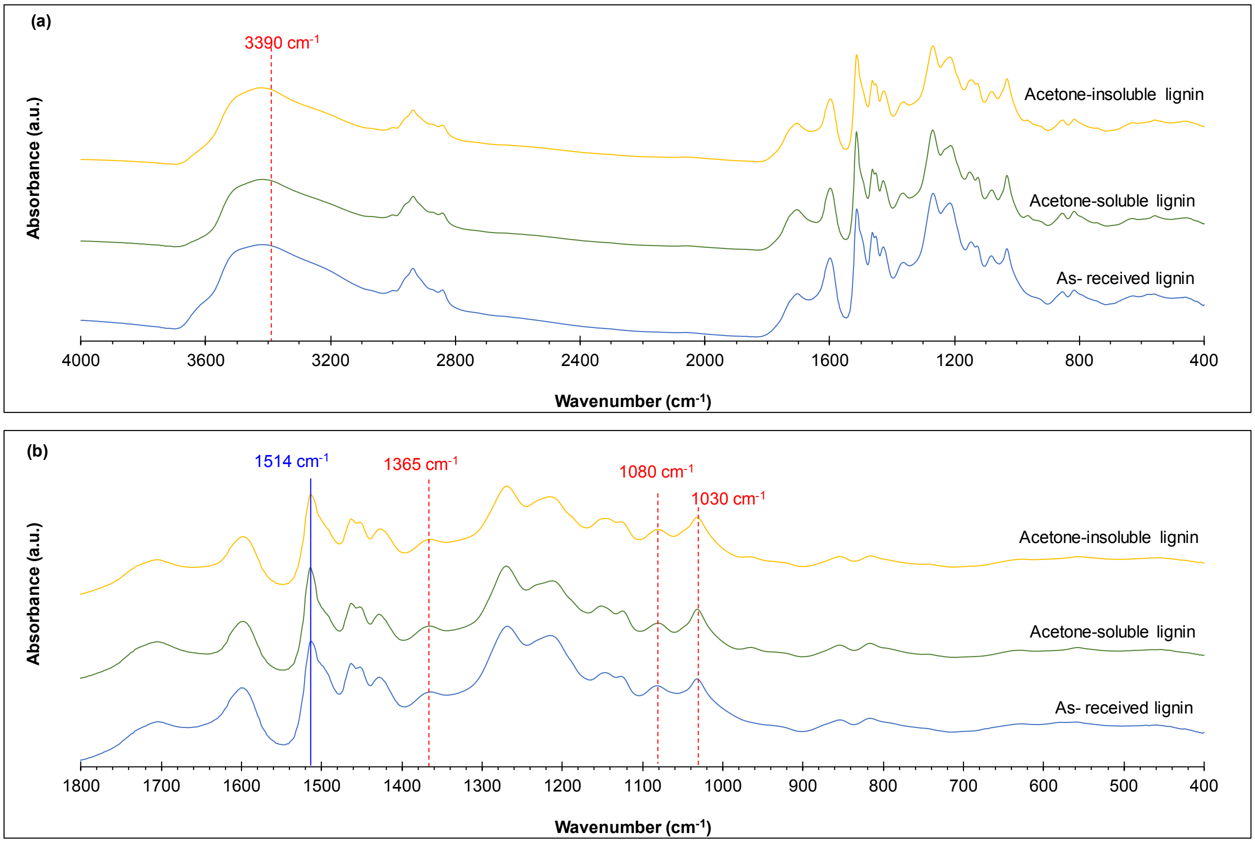

Figure S5. FTIR spectra of as-received lignin: (a) full spectral range; and (b) enlarged view from 1800-400 cm^{-1}.

Table S2. Assignment of FTIR absorption bands for acetone-soluble lignin.

Wavenumber (cm^{-1})	Absorbance peak assignments

Current observations	Previous reports	
3,408	3,440-3,430	O-H stretching in phenolic and aliphatic O-H group
2,940	2,940-2,930	C-H stretching in methyl and methylene group
2,850	2,689-2,880	C-H vibrations of methyl group or methoxy
1,685	1,727-1,690	C = O stretching (in conjugated aldehydes, ketone and carboxylic acids)
1,591	1,594	Aromatic vibration and C-O stretching
1,514	1,515-1,510	Aromatic skeleton vibration
1,460	1,465-1,450	C-H asymmetric bending of methyl and methylene group
1,422	1,428-1,420	C-H vibration of methyl group, aromatic skeleton vibration
1,365	1,370-1,365	C-H deformation in methyl, methoxy (O-CH_3) group
1,365		Phenolic O-H
		C-H deformation in methyl, methoxy groups
1,270	1,270-1,260	Aromatic ring (Guaiacyl moieties) breathing with C = O stretch
1,210	1,200	C-C, C-O and C = O of ester stretching vibrations
1,140-1,120	1,160-1,140	Aromatic C-H in Guaiacyl units
		C-O deformation in primary alcohol
1,082	1,085-1,080	Aliphatic secondary O-H
		C-O deformation in secondary alcohol
1,035	1,035-1,030	Aromatic C-H deformation
		C-O in primary alcohol
		Aliphatic primary O-H
855 and 815	855-852	C-H out-of-plane deformation in positions 2, 5 and 6 of Guaiacyl units

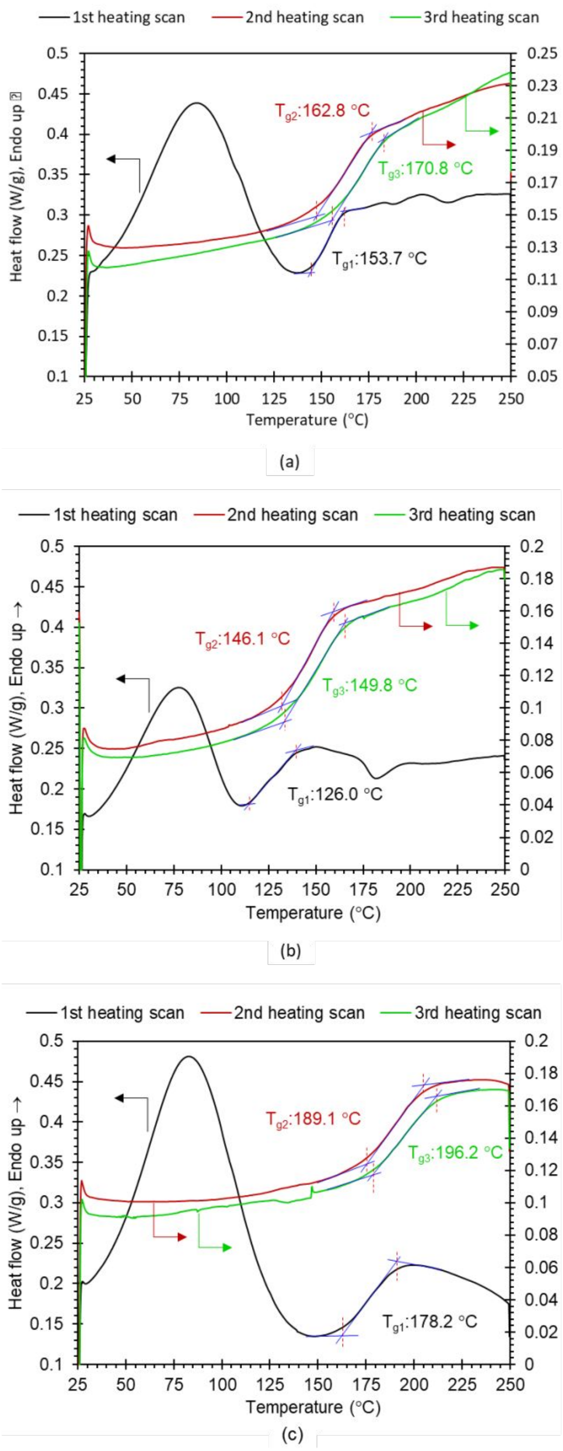

Figure S6. DSC thermograms of (a) as-received lignin, (b) acetone-soluble lignin and (c) acetone-insoluble lignin.

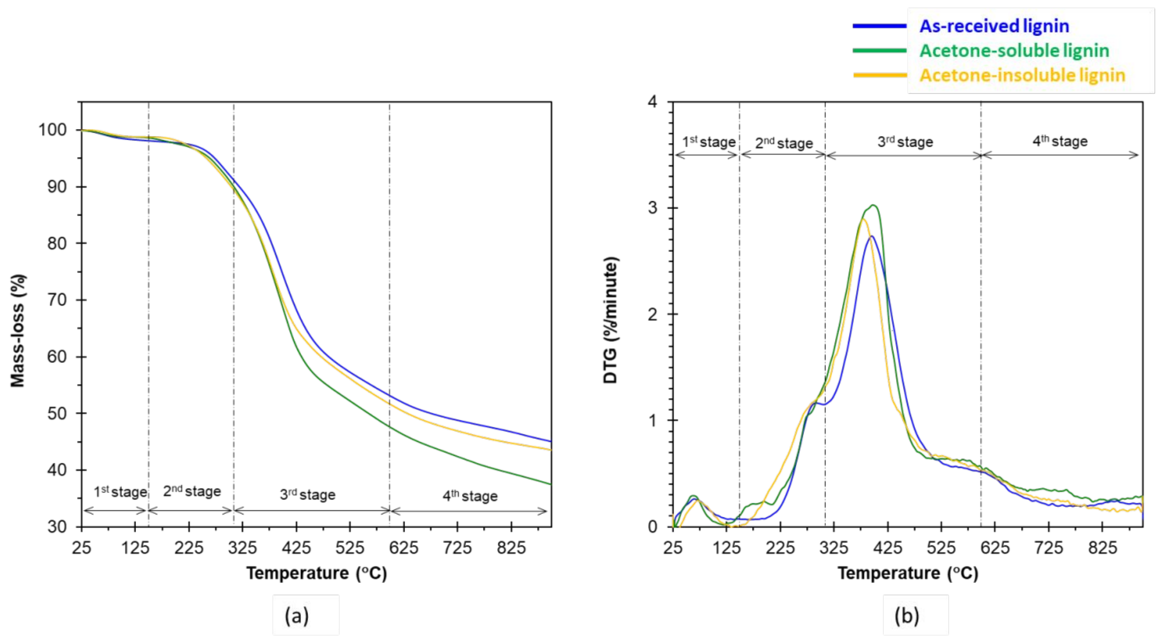

Figure S7. (a) TGA and (b) DTG curves for as-received lignin (blue trace), acetone-soluble lignin (green trace) and acetone-insoluble lignin (yellow trace). The thermal decomposition between 25 °C to 900 °C are divided into four main stages – see text for details.

Table S3. Experiment detail, viscosity and electrical conductivity of acetone-soluble lignin solutions and the observed outcomes during electro-spinning.

Lignin concentrations (wt%)	Volume ratio of acetone/DMSO	Viscosity (Pa·s)	Electrical conductivity ($\mu S \cdot cm^{-1}$)	Observation during electro-spinning
53	1:0 (Neat acetone)	NA	NA	Could not spin, solidification at the needle tip
53	4:1	0.65 ± 0.04	2.01 ± 0.03	Could not spin, solidification at the needle tip
53	3:1	0.54 ± 0.02	2.32 ± 0.04	Could spin for a few seconds.
53	2:1	0.43 ± 0.01	2.37 ± 0.02	Bead-free and continuous electro-spun fibres

45	2:1	0.23 ± 0.00	2.29 ± 0.05	Beaded fibres
48	2:1	0.31 ± 0.03	2.33 ± 0.05	Fibres resembled flat ribbons
58	2:1	0.63 ± 0.04	2.41 ± 0.04	Ribbon-like fibres

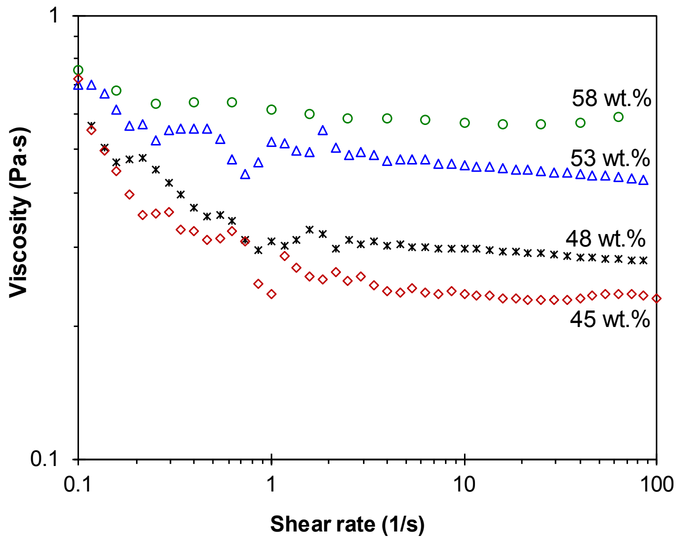

Figure S8. Observed relationship between the viscosity and shear rate for acetone-soluble lignin solutions at specified lignin concentrations.

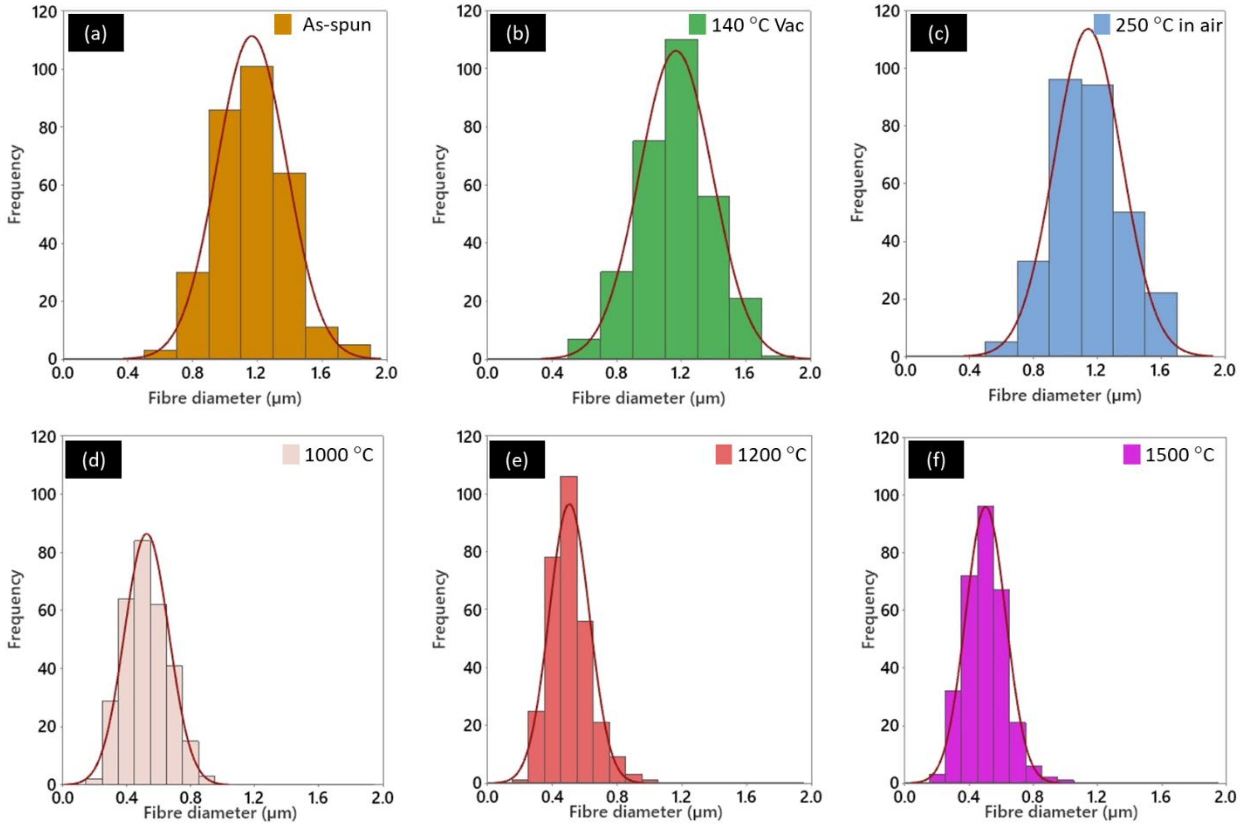

Figure S9. Fibre diameter distribution plots for the acetone-soluble lignin fibres: (a) as-spun; (b) vacuum dried at 140 °C; (c) thermo-stabilised in air at 250 °C; and carbonised in a nitrogen atmosphere at (d) 1000 °C, (e) 1200 °C and (f) 1500 °C.

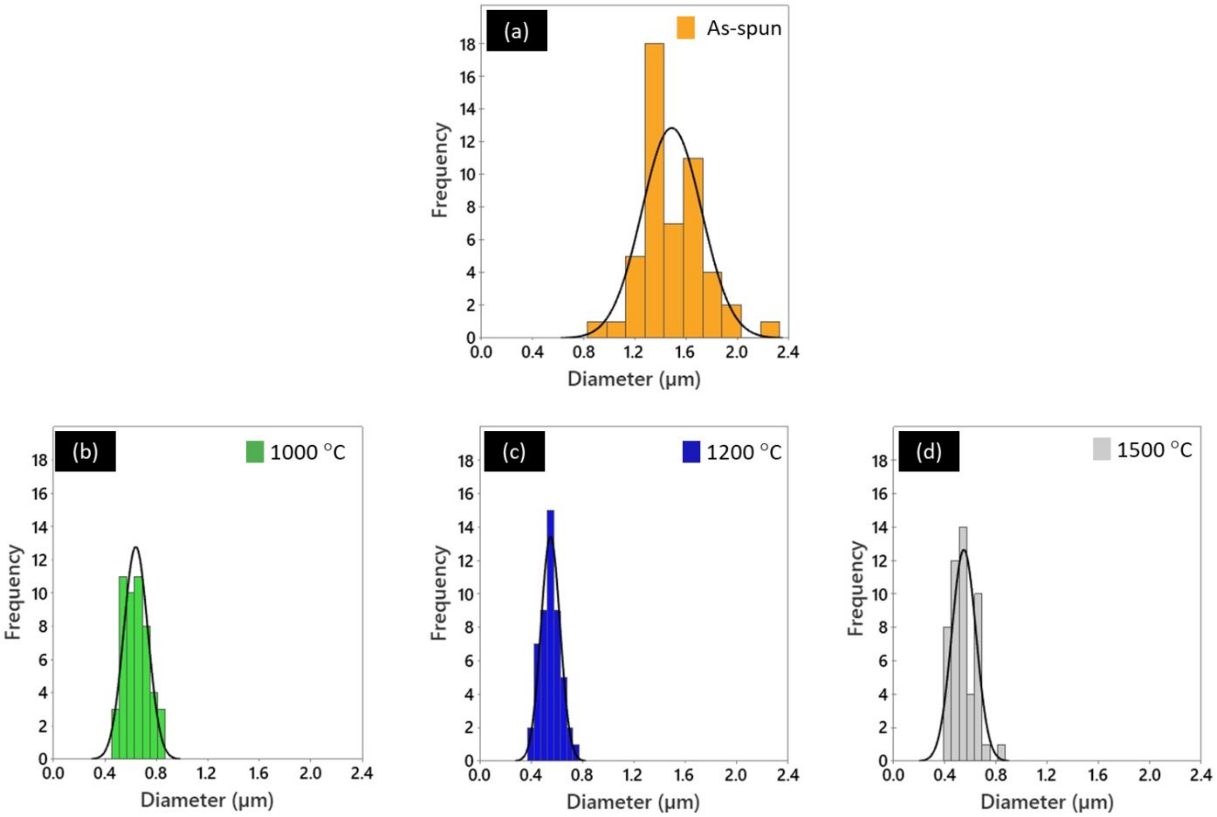

Figure S10. Fibre diameter distribution plots for electro-spun fibres: (a) as-spun fibres aligned fibres; and carbonised aligned fibres at (b) 1000 °C, (c)1200 °C, and (d) 1500 °C.

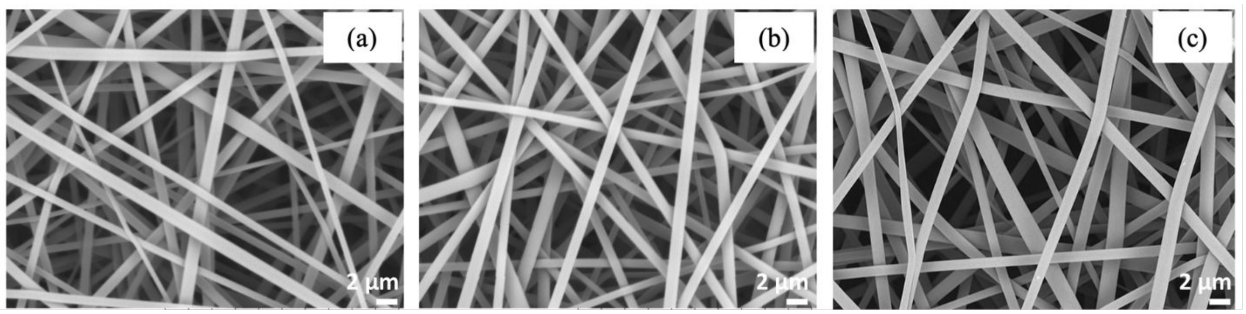

Figure S11. SEM micrographs for the acetone-soluble randomly-oriented electro-spun lignin fibres: (a) as-spun, (b) heat treated in a vacuum oven at 140 °C for 6 hours and (c) thermo-stabilised in air at 250 °C for 1 hours.

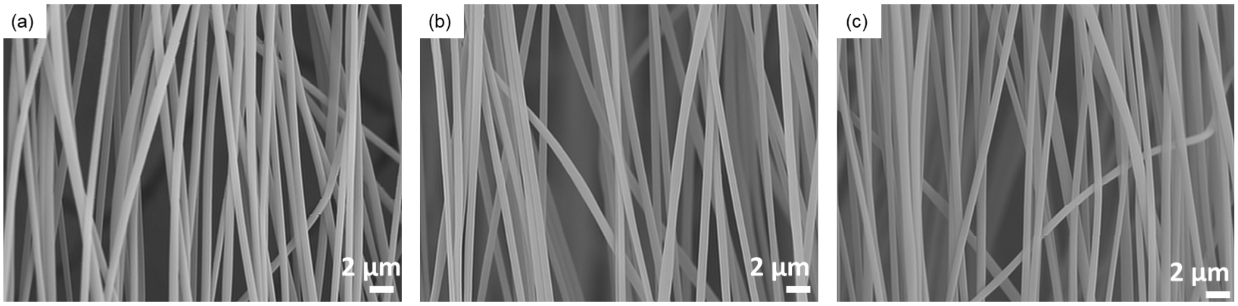

Figure S12. SEM micrographs representing aligned lignin fibres that were carbonised at (a) 1000 °C, (b) 1200 °C and (c) 1500 °C for 1 hours in a nitrogen atmosphere.
